# Optimization of multi-environment trials for genomic selection based on crop models

**DOI:** 10.1007/s00122-017-2922-4

**Published:** 2017-05-24

**Authors:** R. Rincent, E. Kuhn, H. Monod, F.-X. Oury, M. Rousset, V. Allard, J. Le Gouis

**Affiliations:** 1 INRA, UMR 1095 Génétique, Diversité et Ecophysiologie des Céréales, 5 chemin de Beaulieu, 63100 Clermont-Ferrand, France; 2 Université Blaise Pascal, UMR 1095 Génétique, Diversité et Ecophysiologie des Céréales, 63178 Aubière Cedex, France; 3 INRA, MaIAGE, INRA, Université Paris-Saclay, 78350 Jouy-en-Josas, France

## Abstract

**Key message:**

**We propose a statistical criterion to optimize multi-environment trials to predict genotype × environment interactions more efficiently, by combining crop growth models and genomic selection models**.

**Abstract:**

Genotype × environment interactions (GEI) are common in plant multi-environment trials (METs). In this context, models developed for genomic selection (GS) that refers to the use of genome-wide information for predicting breeding values of selection candidates need to be adapted. One promising way to increase prediction accuracy in various environments is to combine ecophysiological and genetic modelling thanks to crop growth models (CGM) incorporating genetic parameters. The efficiency of this approach relies on the quality of the parameter estimates, which depends on the environments composing this MET used for calibration. The objective of this study was to determine a method to optimize the set of environments composing the MET for estimating genetic parameters in this context. A criterion called OptiMET was defined to this aim, and was evaluated on simulated and real data, with the example of wheat phenology. The MET defined with OptiMET allowed estimating the genetic parameters with lower error, leading to higher QTL detection power and higher prediction accuracies. MET defined with OptiMET was on average more efficient than random MET composed of twice as many environments, in terms of quality of the parameter estimates. OptiMET is thus a valuable tool to determine optimal experimental conditions to best exploit MET and the phenotyping tools that are currently developed.

**Electronic supplementary material:**

The online version of this article (doi:10.1007/s00122-017-2922-4) contains supplementary material, which is available to authorized users.

## Introduction

In plant breeding, the best performing varieties are often different from one environment to another. These phenomena are called genotype × environment interactions (GEI). To cope with them, breeders repeatedly phenotype the same varieties in multi-environment trials (METs). However, this approach has economical limitations and screening all materials in all environments is not feasible. Therefore, it would be of great interest to develop models able to predict these interactions. One promising tool to reach this goal is genomic selection (GS), which is a method used in animal and plant breeding to predict genomic breeding values using genome-wide molecular markers (Whittaker et al. 2000; Meuwissen et al. 2001). In a few recent studies, it was proposed to adapt the reference GS models to the GEI context by attributing environment specific effects to the markers (Schulz-Streeck et al. 2013; Crossa et al. 2015), or by modelling environmental covariances (Burgueño et al. 2012). In other studies, environmental covariates were introduced in the GS model (Heslot et al. 2014; Jarquín et al. 2014; Malosetti et al. 2016), which allows predicting in new environments. However, the gain obtained with these models is limited. One likely reason is that the GEI is, in most cases, reduced to linear relationships between varieties and a few environmental covariates, and this cannot allow for the complex interactions between plant development and the environmental conditions.

**Fig. 1:**
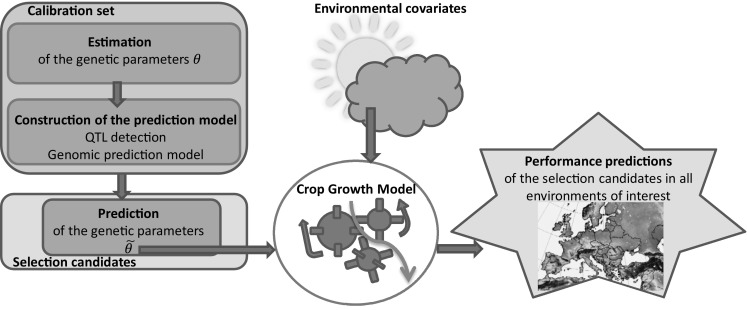
Schematic representation of the CGM-MAS approach. The CGM predicts the performance of the selection candidates in various environments using the (predicted) genetic parameters and the environmental covariates as inputs. The genetic parameters of the calibration set are estimated and used to calibrate a prediction equation (GS models). This equation can then be used to predict the genetic parameters of other genotyped individuals (the selection candidates)

The way plants interact with the environment has long been the subject of refined analyses by ecophysiologists. Their research has allowed to develop crop growth models (CGM) which describe plant development using mechanistic relationships with physiological parameters and environmental covariates as inputs. By definition, the physiological parameters are independent from the environment, but some of them, called the genetic parameters, may depend on the variety. For example, the sensitivity to photoperiod of a given variety is the same for any environment. However, photoperiod can vary from one environment to another which generates GEI, because other varieties can have different photoperiod sensitivities. CGM can be used to predict GEI, since they integrate explicitly both variety characteristics (genetic parameters) and environmental covariates (Chapman et al. 2002; Hammer et al. 2002; Bertin et al. 2010; Bustos-Korts et al. 2016). Once the genetic parameters have been estimated, their genetic architecture can be determined and GS models can be calibrated. The GS model can then be used to predict the genetic parameters of other varieties. These predicted genetic parameters can also be used to predict integrative traits such as yield for these new varieties in new environments by running the CGM (Fig. 1). The interest and feasibility of this approach coupling CGM and genetics have been validated for leaf elongation rate in maize (Reymond et al. 2003; Chenu et al. 2008), fruit quality (Quilot et al. 2005; Prudent et al. 2011), and phenology of various species (White and Hoogenboom 1996; Yin 2005; Nakagawa et al. 2005; Messina et al. 2006; White et al. 2008; Uptmoor et al. 2011; Zheng et al. 2013; Bogard et al. 2014; Onogi et al. 2016). Recently, Technow et al. (2015), Cooper et al. (2016), and Messina et al. (2017) have illustrated the interest of coupling CGM and GS models for predicting and selecting highly integrated traits such as grain yield. One major advantage of their approach and the approach of Onogi et al. (2016) is that the genetic parameters and the marker effects are jointly estimated, and so information can be shared between individuals thanks to the genotypic data.

The approach coupling CGM and marker-assisted selection (CGM-MAS) is also called gene-based modelling or QTL-based modelling (see Fig. 1). In CGM-MAS, the efficiency of genome-wide association studies (GWAS) and GS for the genetic parameters depends on the composition of the calibration set, the relevance of the crop model, and the quality of the parameter estimates. Considering the number of genetic parameters involved in crop models and the way they are entangled in complex processes, it is quite clear that huge amounts of data are required to estimate parameters and that the high-throughput phenotyping tools under development such as drones (UAV) and phenotyping platforms will considerably help.

Some genetic parameters can be estimated almost directly by measuring simple traits on phenotyping platforms (Reymond et al. 2003; Yin 2005). The other genetic parameters are estimated by adjusting the CGM outputs to the observations of more integrative traits. In this case, the inference of the parameters can be done thanks to brute-force algorithm (Bogard et al. 2014), more sophisticated exploration algorithms (Wallach et al. 2011; Klein et al. 2012) or Bayesian inference (Makowski et al. 2002; Van Oijen et al. 2005; Iizumi et al. 2009; Dumont et al. 2014). The quality of the parameter estimates highly conditions QTL detection power (Wang 2008; Teyssèdre et al. 2012; Rincent et al. 2014) and GS accuracy (Daetwyler et al. 2008; VanRaden 2008) through the error variance.

A fundamental issue for data collection and parameter inference is the choice of the experimental design. Most literature on the design of experiments in plant breeding concentrates on the within-trial allocation of varieties to plots (Piepho and Williams 2006; Butler et al. 2014). When designing METs to estimate genetic parameters, however, the major question is to determine in which pedoclimatic conditions the varieties need to be phenotyped to provide the best estimates. For example, if day length plays a major role in the CGM behavior, one can expect that experimental designs capturing important variability of day lengths will be more efficient than others for estimating the corresponding genetic parameters. The importance of the definition of the experimental design to estimate model parameters has been discussed in some studies (Wöhling et al. 2013; Dumont et al. 2014). The definition of optimal MET design is thus a key point, which must be based on sound statistical approaches.

A few studies have tackled explicitly the design of MET (see Talbot Chapter 10 on resource allocation for selection systems in Kempton and Fox 1997). More recently, an interesting approach was proposed to optimize experimental designs to calibrate hydrological model (Leube et al. 2012). Developing such new tools in the context of CGM-MAS is a main current necessity of great interest.

The main objective of this study was to develop a statistical criterion to optimize the set of environments composing the MET before collecting data in the context of CGM-MAS. The designs sampled with this criterion should allow for the most efficient calibration of the crop model for a whole collection of varieties. The high quality of the genetic parameter estimates obtained with the optimal experimental design should in turn increase CGM-MAS efficiency (QTL detection power and prediction accuracy). These approaches were tested on wheat phenology (heading time), for which reference crop models exist (Jamieson et al. 1998b; Keating et al. 2003), using simulated and real data sets. This trait is influenced by temperatures and day length, so we focused on the definition of optimal combinations of locations and sowing dates. A Bayesian inference approach was used to estimate the genetic parameters of crop models using integrative phenotypes.

## Materials and methods

Our objective is to determine an optimal set of environments (multi-environment trials, MET) for the estimation of genetic parameters. When the optimal MET must be determined, only the crop model and the possible sites are known. There is not yet any measurement available on the environmental covariates of the CGM for the year to come, but we consider that the measurements in the past years can be used to approximate them.

Formally, we consider that *I* genotypes have to be phenotyped in *Z* environments with *K* replications per environment. We denote by  the collection of all the environments considered in the study:  $= \;\left\{ {E_{j} ,\; 1 \le j \le J} \right\},$ where $E_{j}$ is the vector of environmental covariates of environment *j* required for the crop model to run, and *J* is the total number of possible environments. We define an MET of size *Z* as a subset of  composed of *Z* environments. For a given MET *d*, we denote by $E_{d}$ the joint vector of the environmental values $E_{j} ,$ $j \in$  being the set of indices of the environments composing it.

### Statistical model

We assume that the observations are the sum of the crop model output and an error term. Thus, the statistical model is:

1 $$Y_{ijk} \; = \;f\left( {\theta_{i} ,\;E_{j} } \right)\; + \;e_{ijk} ,{\text{ for}}\;1 \le i \le I,\;1 \le j \le J\;{\text{and}}\;1 \le k \le K,$$

where $Y_{ijk}$ is the scalar value of genotype *i* in environment *j* and replication *k*, $f$ is the function corresponding to the crop model (usually non-linear), $\theta_{i}$ is the *p*-dimensional vector of genetic parameters for variety *i*, and $e_{ijk}$ is an additive error term. The residuals ($e_{ijk}$) are assumed to be independent, normally distributed, centered with variance $\sigma_{e}^{2}$ for sake of simplicity, but heteroscedasticity and non-normal distributions could be used as well if required.

For a given MET *d* of size *Z*, we denote by $Y_{i}^{d}$ the vector of output values $Y_{ijk}$ for $1 \le k \le K$ and $j \in$ .

In the present study, we estimate the parameters ($\theta_{i} ,\;1 \le i \le I,\;\sigma_{e}^{2}$) of model (1) by a Bayesian inference algorithm applied to the phenotypes collected in the MET *d*. Prior distributions are given low information levels: uniform distributions for $\theta_{i}$ with bounds defined thanks to literature or expert knowledge, and inverse Gamma distribution for $\sigma_{e}^{2}$. Of course, if the MET is more complex than the one described here, one can adjust model (1) for a better inference adapted to these situations (for example by taking into account block effects, or by introducing heteroscedasticity as done in the present study for the real data set).

### Definition of the criterion OptiMET used to optimize MET

The OptiMET criterion is inspired by optimal Bayesian design (see Atkinson and Donev 1992) and adapted from the study of Leube et al. (2012) in the context of Bayesian model averaging for hydrological models. Our objective is to define a relevant MET which is able to differentiate both between two different parameter values leading to two different observation values and between two different observation values corresponding to two different parameter values. Therefore, we require at least that the MET is built, such that the crop model generates distant outputs for distant genetic parameter vectors. We will propose a criterion to determine a set of environments (MET) satisfying this condition.

Since we do not know the values of parameter vector $\theta$ when determining the MET, we will consider a huge finite number of possible a priori values, standing for the parameter vector distribution across varieties. Therefore, we consider a sample of size *m* denoted by $\left( {\theta_{u} } \right)_{1 \le u \le m}$ which is a finite size representation of the possible continuous distribution of the parameters. These *m* genetic parameter vectors can be chosen based on expert knowledge or on literature (it is most of the time possible to define at least lower and upper bounds). The distance between any two parameter vectors $\theta_{u}$ and $\theta_{v}$ is defined by:

$${\text{dist}}\left( {\theta_{u} ,\theta_{v} } \right) = \left[ {\mathop \sum \limits_{s = 1}^{p} \left( {\frac{{\theta_{us} - \theta_{vs} }}{{M_{s} - m_{s} }}} \right)^{2} } \right]^{1/2} ,$$

where $\theta_{us}$ and $\theta_{vs}$ are the sth component of $\theta_{u}$ and $\theta_{v} ,$ respectively, and $M_{s}$ and $m_{s}$ are the maximal and minimal value for the sth component of $\theta$ determined by expert knowledge or using the literature.

For a given candidate MET *d*, we denote by $L^{d}$ the matrix of size *m* × *m*, in which the element $L_{uv}^{d}$ is computed following Leube et al. (2012):

$$L_{uv}^{d} \; = \;\frac{1}{{\left( {4\pi \sigma_{e}^{2} } \right)^{{n_{y} /2}} }}\exp \left( { - \frac{1}{{4\sigma_{e}^{2} }}\left( {\Delta_{uv}^{d} } \right)^{t} \Delta_{uv}^{d} } \right),$$

where $n_{y}$ is the number of observations for a given variety ($n_{y} = Z \times K,$) and $\Delta_{uv}^{d} = (f\left( {\theta_{u} ,E_{j} } \right) - f\left( {\theta_{v} ,E_{j} } \right), j \in$ ). The quantity $L_{uv}^{d}$ corresponds to the likelihood of the parameter vector $\theta_{u}$ given the synthetic noise-free data ($f\left( {\theta_{v} ,E_{j} } \right), j \in$ ) (for more details, see Leube et al. 2012, Appendix B).

The matrix ($L_{uv}^{d}$) is normalized by computing the weight matrix $W_{uv}^{d} = \frac{{L_{uv}^{d} }}{{\sum u L_{uv}^{d} }}$.

We define the value of the criterion OptiMET for a given MET *d* by: ${\text{OptiMET}}^{d} = \mathop \sum \nolimits_{u,v = 1}^{m} \left( {{\text{dist}}\left( {\theta_{u} ,\theta_{v} } \right)\; \times \;W_{uv}^{d} } \right).$ The optimal design denoted by *d*_opt_ is the one that minimizes OptiMET. Indeed minimizing OptiMET results in maximizing the distance between the outputs of the CGM for two genetic parameters vectors that are distant, i.e., minimizing the corresponding coefficient in the weight matrix *W*.

### Case study: MET optimization for the estimation of Sirius CGM phenology parameters

**Fig. 2:**
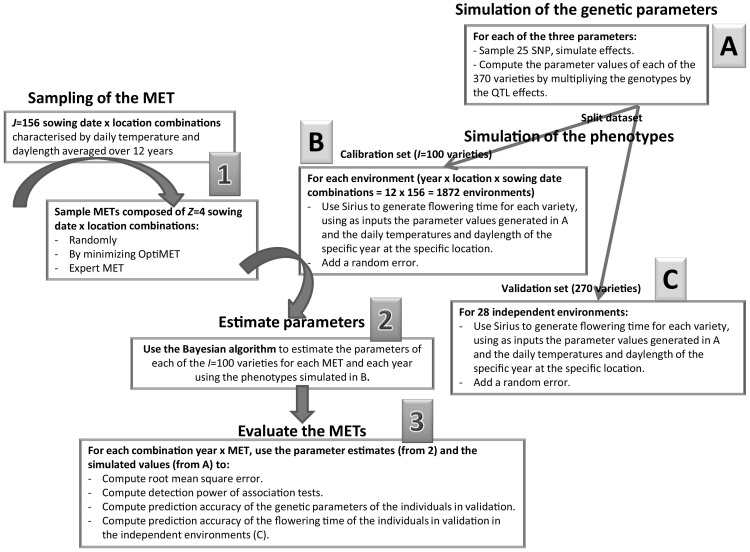
Process used to compare by simulation the efficiency of different METs. Different MET sampling strategies are compared and evaluated for their efficiency to estimate the genetic parameters, detect QTL in the calibration set, and predict performance of the validation set in independent environments

Many strategies to sample MET (combinations of locations and sowing dates in this case study) can be compared (Fig. 2, box 1). In this paper, we concentrate on three of them: random sampling, OptiMET-optimal sampling, and sampling based on expert knowledge. Wheat heading time was used as a case study. This trait is key to plant adaptation to new environments, and it has been intensively studied and modelled.

To evaluate the efficiency of OptiMET to optimize the composition of MET, we have tested it both with simulations and real data. In the simulation part, we considered that phenotypes were generated according to model (1). In a second part, OptiMET was tested on a real data set, to evaluate its robustness.

#### Sirius crop model

Sirius is a reference crop model to simulate wheat development (Jamieson et al. 1998b). Its relevance to simulate accurately the development of crops was validated in wide range of conditions including Europe, New Zealand, Australia, and USA (Semenov et al. 1996; Jamieson et al. 1998a, b; Jamieson and Munro 2000; Jamieson and Semenov 2000; Brooks et al. 2001). The phenology model is described in He et al. (2012). Briefly, the development of wheat from sowing to heading is modelled in three phases. The first phase, from sowing to emergence, is simulated as a fixed thermal time duration. In a second phase, from crop emergence to flag leaf appearance, flag leaf appearance successively integrates the effects of vernalization and photoperiod coupled with the rate of leaf emission (phyllochron). The last phase, from leaf ligule appearance to heading, is purely proportional to the phyllochron.

It has been shown that the processes of leaf appearance rate, and sensitivity to vernalization and to photoperiod have important genetic variability and strongly influence flowering time (He et al. 2012; Martre et al. 2015a). For these reasons, we defined as genetic parameters $\theta$ the three main parameters (*p* = 3) driving these processes: the response of vernalization rate to temperature (VAI), the day length response of leaf production (SLDL), and the phyllochron (Phyl). In addition to these three parameters, the model requires daily average temperature and day length to run.

#### Computation of OptiMET

When computing OptiMET to determine an optimal MET (combination of sowing dates and locations), the climatic conditions that will occur at each location during the experiment are unknown (except day length). However, as daily temperatures are required to compute OptiMET, it was approximated by the average of daily temperature across a number of years sufficient to get stable averages. This virtual year characterized by daily temperatures averaged over 12 years is further referred to as the “average year”.

To compute OptiMET, we discretized each parameter interval into ten regularly spaced values and used the *m* = 10^3^ = 1000 combinations for the genetic parameter vectors.

#### Parameter estimation using MCMC algorithm

The prior distributions of the genetic parameters (*VAI*, *SLDL,* and *Phyl*) were defined as uniform distributions with minimum and maximum fixed using knowledge of experts and found in the literature (He et al. 2012; Martre et al. 2015a):

$$VAI\sim U\left( {0,0.01} \right),$$

$$SLDL\sim U\left( {0,1} \right),$$

$$Phyl\sim U\left( {80,120} \right).$$

The prior distribution of $\sigma_{e}^{2}$ was defined as a non-informative inverse Gamma distribution (with shape and scale parameters of 4 and 0.2, respectively). For the simulation study, the same residual variance $\sigma_{e}^{2}$ was attributed to all environments. For the study on real data, the residual variances were specific to each environment to model heteroscedasticity.

To generate the posterior distributions, we have used as MCMC algorithm a hybrid Gibbs sampler by block which updates in turn three coordinates at a time for $\theta_{i}$ (the parameter values of VAI, SLDL, and Phyl of each variety in turn) and then $\sigma_{e}^{2}$, through a Metropolis–Hastings step using as proposal a Gaussian distribution centered on the previous value of the chain. 20,000 iterations were generated and the first 1000 were discarded (burn-in). Parameter estimates were defined as the mode of the posterior distributions. All scripts were written in R 2.14.0 and can be made available upon request.

### Evaluation of the criterion OptiMET using simulations

#### Procedure overview

The procedure used to compare the efficiency of different MET to estimate the genetic parameters is illustrated on Fig. 2. The objective is to evaluate the efficiency of OptiMET to optimize MET (combinations of locations and sowing dates) for the estimation of the three phenological parameters. Real genotypes were used to simulate QTL (Fig. 2, box A) for each genetic parameter. The parameter values were then computed for each variety. The varieties were split into two data sets: a calibration set and a validation set. The crop model Sirius was then used to generate phenotypes for the individuals of the calibration set in each considered environment for various years (Fig. 2, box B). In parallel to this, various strategies were used to sample MET of a given size among all the possible environments (Fig. 2, box 1). These strategies were: random sampling, sampling by minimizing OptiMET, and choosing an “expert MET” based on expert knowledge. The phenotypes generated for each of these MET for each specific year were used to estimate the parameters for each specific year independently (Fig. 2, box 2). For each MET and each year, we thus obtained parameter estimates. To evaluate the estimation efficiency of each MET, for each specific year, we computed root-mean-square errors (RMSE) of these estimates (as the true parameter values are known in simulation setting), detection power of association tests, and prediction accuracy of the parameter values of the individuals in validation (Fig. 2, box 3). Finally, for each MET and each specific year, we used the parameter predictions of the individuals in validation to predict using the CGM their heading time in independent environments representing the variability of French wheat production environments (as defined below) and computed the corresponding heading time prediction accuracies. The efficiency of the different MET was computed for each specific year independently to evaluate the stability of the different MET sampling strategies over years. It was not tried here to combine different years in a same MET, but this would be in practice possible.

#### Environments

In this section based on simulations, MET were composed of four location × sowing date combinations (*Z* = 4) and sampled among 156 possible sowing date x location combinations (*J* = 156). These 156 combinations are composed of 39 locations (supplementary information Fig. S1) spread in France combined with four sowing dates including three winter sowing dates (15th September, 15th October, 15th November) and one spring sowing date (15th March).

Twenty-eight independent environments were used to validate heading time prediction accuracy of the validation set (Fig. 2, box C). These 28 environments are representative of usual wheat growing conditions in France (Agreste 2016). They are the combinations of seven locations (representing the main regions in France where wheat is grown, supplementary information Fig. S1), two sowing dates (15th October and 15th November) with the climatic conditions of two specific growing seasons (2010/2011 and 2011/2012).

#### Phenotype simulation

Simulations were based on the real genotypes of a panel of 370 accessions from the INRA bread wheat core collection which was defined to represent worldwide wheat diversity (Balfourier et al. 2007) and with a large variability of growth habit (Rousset et al. 2011). All these lines were genotyped with an Affymetrix Axiom 280 K SNP array developed in the frame of the BreedWheat project (Rimbert et al. in preparation). After filtering for quality and homozygosity, the genotypes consisted of 20,713 SNP with known genetic positions.

To simulate the genetic architecture of the three genetic parameters, 25 SNP were sampled independently for each parameter and defined as QTL. Their effects followed geometric series as defined in Lande and Thompson (1990). The QTL effects were then rescaled, such that the genetic parameters took values with biological relevance (i.e., in the ranges defined above). The 75 SNP defined as QTL were then removed from the data set. At this step, each variety was defined by a vector of three parameters. The 370 accessions were then split in two data sets: 100 randomly sampled composed the calibration set and the 270 remaining the validation set. The sampling of the calibration set was done only once because of the computational burden of the simulation procedure.

To simulate phenotypes of the 100 individuals composing the calibration set in each environment (sowing date × location × year = 4 × 39 × 12 = 1872), Sirius was run for each variety with the environmental covariates of the specific year (daily temperature and day length) and the genetic parameter values (computed using the simulated QTL) as inputs. Residual errors were added to Sirius outputs using a centered normal distribution with a standard deviation relevant to mimic real experimental conditions (2 days).

#### Multi-environment trials sampling

Different sampling approaches have been used:

- Random MET. For each year (from 2003 to 2014), 10 MET of size *Z* = 4 were randomly sampled with 0, 1, 2, 3, or 4 winter sowings (and as a consequence 4, 3, 2, 1, or 0 spring sowings). This resulted in 120 random MET in total.
- An “expert MET”. This MET was defined using the knowledge of experts with the objective of estimating the different earliness components. It is composed of three locations chosen to get a North–South gradient (Mons-en-Chaussée, Versailles and Clermont-Ferrand) and, as a result, a photoperiodic gradient, with a winter sowing date (15th November) at each location, and an additional spring sowing date in Clermont-Ferrand (15th March). This combination of a winter and a spring sowing at the same location is supposed to capture efficiently the effect of vernalization.
- The MET minimizing OptiMET. To compute OptiMET, we considered environmental covariates (daily temperature and day length) averaged over 12 years (from 2003 to 2014). Considering the huge amount of environments, it was not possible to determine analytically the MET *d*_opt_ minimizing OptiMET. For this reason, we used an exchange algorithm: at each step, the random exchange of one environment included in the MET with one environment excluded was accepted if OptiMET decreased and was rejected otherwise. 3000 iterations were sufficient to reach a minimum, and we checked that the final MET was not a local optimum by running the exchange algorithm in parallel with four different initializations.

#### Evaluation of the efficiency of the METs

To compare the efficiency of each MET, the normalized root-mean-square errors (NRMSE) of the parameter estimates were computed for $1 \le s \le p$, for each MET and each year of experiment as follows: ${\text{NRMSE}}\left( {\theta_{s} } \right) = \left[ {\frac{1}{I}\mathop \sum \nolimits_{i = 1}^{I} \left( {\frac{{\hat{\theta }_{is} - \theta_{is} }}{{M_{s} - m_{s} }}} \right)^{2} } \right]^{1/2} ,$ with *I* = 100, the number of genotypes, and where $\hat{\theta }_{is}$ is the estimate of parameter $\theta_{is}$ using the MET considered equal to the mode of the posterior distribution, and $M_{S}$ and $m_{S}$ are the maximal and minimal values of $\theta_{is}$ with $1 \le i \le I$.

We have also compared the METs efficiency (1) to detect QTL, and (2) to predict the parameters of independent varieties. For (1), for each of the three genetic parameters, a QTL was considered to be detected if at least one marker located at less than 1 cM from the simulated QTL was significantly associated (*P* value below a threshold of 0.05/25). The statistical model of Yu et al. (2006) with a random polygenic effect but no structure effect was used to test for associations. The covariance matrix of the random polygenic effect was estimated with the genotypic data (after removing the 75 SNP defined as QTL) using the estimator of VanRaden (2008). The detection power obtained with the different METs could then be compared. For (2), a classical G-BLUP model (Habier et al. 2008; Zhong et al. 2009) was used to predict the parameter values of the 270 individuals composing the validation set using the same kinship matrix than for the QTL detection. The prediction accuracy could then be computed as the correlation between predictions and simulated parameter values. Finally, these predicted parameter values could be used to predict heading time of the 270 varieties in the 28 independent environments using Sirius crop model (Fig. 2). Root-mean-square error (RMSE) and prediction accuracy of heading time were then computed to compare the MET. Prediction accuracy was defined as the correlation between predicted and simulated heading time.

### Evaluation of OptiMET on a real data set

#### Description of the data set

To further evaluate the efficiency of OptiMET, a test on a real data set was also performed. In this data set, 121 varieties (*I* = 121) adapted to French environments with contrasted phenologies were phenotyped for heading date in 26 environments (*J* = 26, see Table 1) without replication (*K* = 1). These environments were in the western part or northern part of France, and sowing dates ranged from 17th of October to 14th of April.

**Table 1:** Environments in which heading date of the 121 varieties was observed. In this real data set, 121 varieties were phenotyped for heading date in 26 environments with sowing dates ranging from October to April

2008/2009		2009/2010	
Sowing date	Location	Sowing date	Location
17/10/2008	Allonnes	23/10/2009	Mons-en-Chaussée
20/10/2008	Mons-en-Chaussée	28/10/2009	Clermont-Ferrand
20/10/2008	Le Moulon	28/10/2009	Louville
22/10/2008	Auchy	29/10/2009	Clermont-Ferrand
23/10/2008	Villiers-le-Bâcle	29/10/2009	Maule
29/10/2008	Montroy	29/10/2009	Caussade
12/11/2008	Clermont-Ferrand	30/10/2009	La Minière
20/11/2008	Clermont-Ferrand	25/11/2009	Villiers-le-Bâcle
12/12/2008	La Minière	14/12/2009	Clermont-Ferrand
24/12/2008	Mons-en-Chaussée	15/12/2009	Clermont-Ferrand
05/01/2009	Clermont-Ferrand	23/02/2010	Clermont-Ferrand
25/02/2009	Clermont-Ferrand	04/03/2010	Mons-en-Chaussée
16/03/2009	Mons-en-Chaussée		
14/04/2009	Mons-en-Chaussée		

#### MET sampling

METs of 4, 6, or 8 environments (*Z* = 4, 6 or 8) were sampled by minimizing OptiMET, or randomly, or with a reasoned strategy. In the reasoned strategy, we imposed that all sowing periods were represented in the MET. These four periods were October, November/December, January/February, and March/April. For each MET size (4, 6, and 8), we chose to sample 40 random trials and 40 reasoned trials. For the computation of OptiMET, environmental covariates were required. For this reason, we have used the daily average temperature of the closest meteorological station averaged over 11 years (2001–2014, excluding the years of experiment: 2008–2010).

#### Evaluation of the efficiency of the METs

A direct evaluation of the root-mean-square error of the parameter estimates was not possible in this case, because the real parameter values are unknown. For this reason, we have estimated the parameters using the 26 environments simultaneously using the Bayesian algorithm and used these estimates, denoted by $\theta_{is}^{*}$ for $1 \le i \le I = 121$ and $1 \le s \le p = 3$ as references. The use of these 26 environments to generate reference estimates seems reasonable as there are only three parameters, and the 26 environments are well adapted to estimate phenological parameters. Given these estimates, we can now consider criteria to evaluate the efficiency of the different METs to capture the information that is present in the whole data set.

Therefore, we considered two complementary criteria:

- First, the normalized root-mean-square error computed for $1 \le s \le p$ as: ${\text{NRMSE}}^{*} \left( {\theta_{s} } \right) = \left[ {\frac{1}{I}\mathop \sum \nolimits_{i = 1}^{I} \left( {\frac{{\hat{\theta }_{is} - \theta_{is}^{*} }}{{M_{s}^{*} - m_{s}^{*} }}} \right)^{2} } \right]^{1/2} ,$ with *I* = 121, and where $\hat{\theta }_{is}$ is the estimate of parameter $\theta_{is}$ using the MET considered and equal to the mode of the posterior distribution, and $M_{s}^{*}$ and $m_{s}^{*}$ are the maximal and minimal values of the reference parameter estimates $\theta_{is}^{*}$.
- Second, the normalized posterior square error (NPSE), defined for $1 \le s \le p$ as: ${\text{NPSE}}\left( {\theta_{s} } \right) = {\mathbb{E}}_{\pi } \left[ {\frac{1}{I}\mathop \sum \nolimits_{i = 1}^{I} \left( {\frac{{\theta_{is} - \theta_{is}^{*} }}{{M_{s}^{*} - m_{s}^{*} }}} \right)^{2} } \right],$ where $\pi$ is the posterior distribution of $\theta_{is}$ conditionally to the data, ${\mathbb{E}}_{\pi }$ the corresponding expectation, and *I* = 121. Indeed, since the whole posterior distribution is available in our Bayesian context, we can also evaluate the precision of the estimation through this integrated quantity which can be seen more like a variance. However, it is not possible to compute it analytically. Therefore, we calculated an empirical version using the last realizations of ($\theta_{is}$) resulting from the Metropolis–Hastings (MH) algorithm. More precisely, let us denote by $\left( {\theta_{is}^{k} } \right)_{1 \le k \le K}$ the *K* last realizations of the MH algorithm for $1 \le i \le I$ and $1 \le s \le p$. We computed the following quantities $\frac{1}{I}\mathop \sum \limits_{i = 1}^{I} \left[ {\frac{1}{K}\mathop \sum \nolimits_{k = 1}^{K} \left( {\frac{{\theta_{is}^{k} - \theta_{is}^{*} }}{{M_{s}^{*} - m_{s}^{*} }}} \right)^{2} } \right]$ using *K* = 19,000.

For each MET, both criteria were averaged over the three parameters, leading to $\overline{{{\text{NRMSE}}^{*} }}$ and $\overline{\text{NPSE}}$.

## Results

### Evaluation of the criterion OptiMET using simulations

#### Root-mean-square error of the parameter estimations obtained with the different METs

**Fig. 3:**
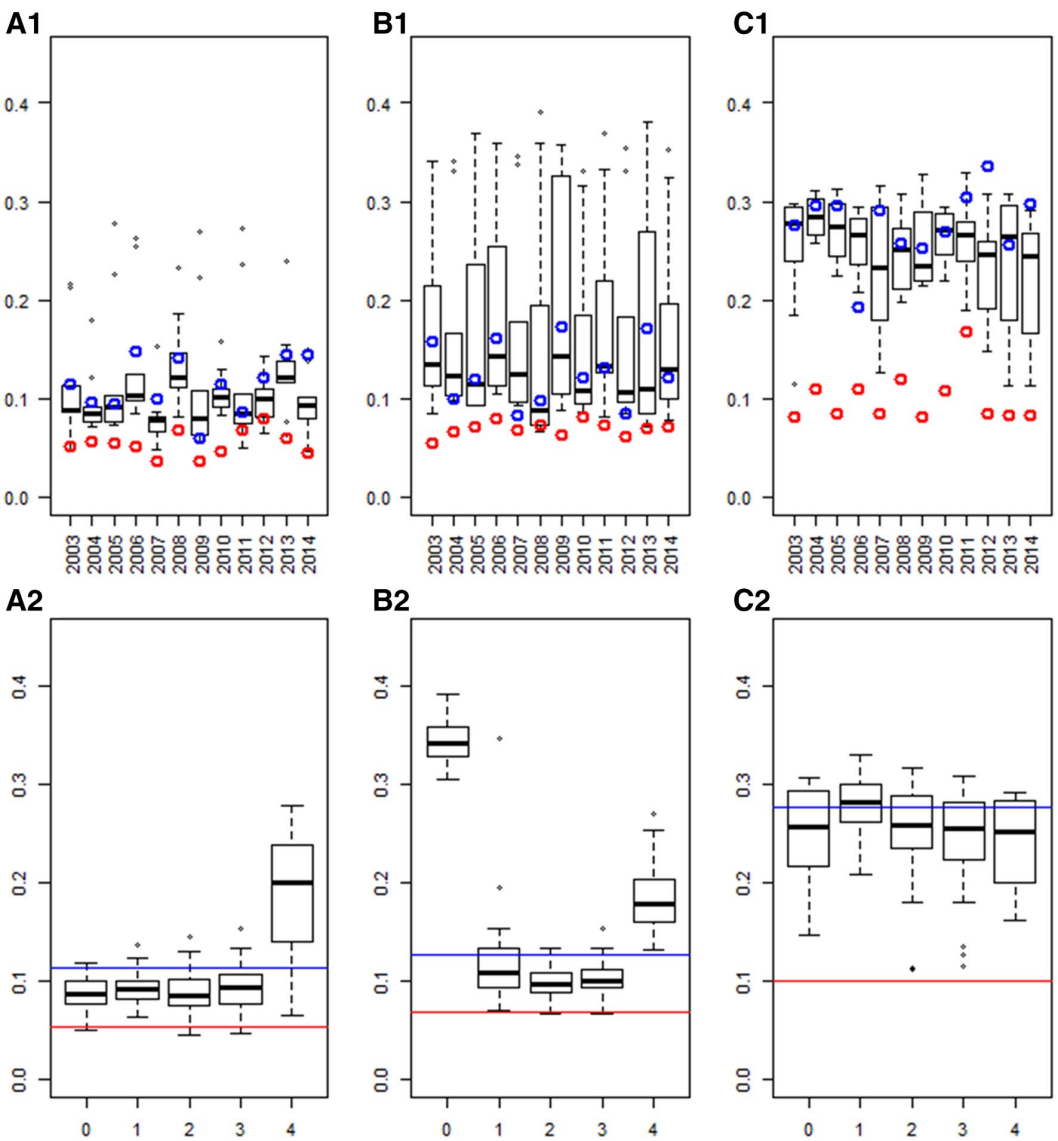
Normalized root-mean-square error (NRMSE) of the parameter estimates of different METs: 120 randomly sampled (*boxplots*), one expert MET (*blue points* for specific years and blue lines for the average across years), and the OptiMET MET (*red points* for specific years and *red lines* for the average across years). The results are presented for each of the 12 years of experiment (**a1**, **b1,** and **c1**), or for each of the random sampling strategy (**a2**, **b2,** and **c2**, number of winter sowings on the *x-axis*), for VAI **a**, SLDL **b,** and Phyl **c** (color figure online)

For each MET (location × sowing date), the NRMSE of the model parameter estimates using the climatic conditions of each of the 12 years were computed (Fig. 3). The NRMSE of randomly sampled METs were highly variable for the three model parameters. For example, NRMSE of parameter Phyl ranged from 0.12 to 0.30 (which corresponds to RMSE of 4.6–12 degree-days) when the experiment was simulated with the climate of year 2013. NRMSE of the expert MET were sometimes higher and sometimes lower than those of the random METs. The MET sampled with OptiMET most of the time resulted in lower NRMSE than random and expert METs, with the exception of parameter SLDL in year 2008 and parameter VAI in years 2011 and 2012. The NRMSE reduction was higher for Phyl than for VAI and SLDL. The ratio between winter and spring sowings of the random MET influenced NRMSE. For VAI, random METs resulted in low NRMSE on average when at least one spring sowing was sampled (Fig. 3a2). For SLDL, the best random METs were those with at least one sowing date of each period (winter and spring). The worst case was when four spring sowings were sampled (Fig. 3b2). For Phyl, there was no clear difference between the different options and all winter/spring sowings resulted in high NRMSE on average (Fig. 3c2). The OptiMET MET did better than the different winter/spring sowing combinations used for random sampling on average.

#### QTL detection power for the parameter estimates obtained with the different METs

**Fig. 4:**
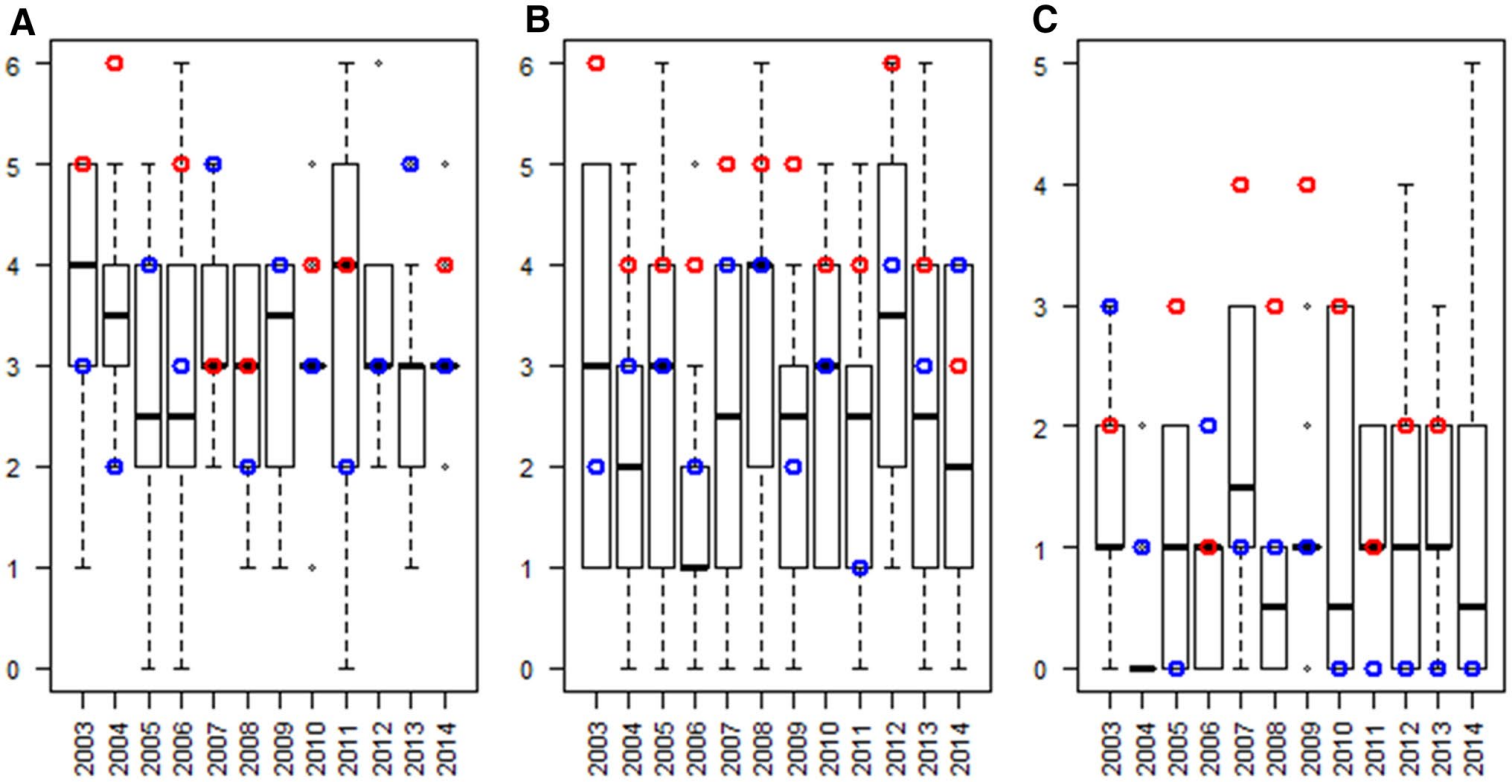
Number of QTL detected in the different METs: 120 randomly sampled METs (*boxplots*), one expert MET (*blue points*), and the OptiMET MET (*red points*). The results are presented for each of the three parameters: VAI **a**, SLDL **b,** and Phyl **c**, for each year of experiment (color figure online)

The second criterion used to compare the different METs is the QTL detection power for the parameter estimates (Table 2). Detection power was low for all METs, with a maximum of 18% for SLDL, which corresponds to 4.5 detected QTL (among the 25 QTL simulated). Power was higher for VAI and SLDL (10.1–18.0%) than for Phyl (3.0–10.7%). On average, power was higher when using the OptiMET MET than the expert or random METs. These trends were consistent across years with the exception of 2007 for VAI, 2014 for SLDL, and 2003 and 2006 for Phyl where the expert MET did better than the OptiMET MET (Fig. 4).

**Table 2:** Average QTL detection power (%) in the different METs. For each of the three parameters (VAI, SLDL, and Phyl), detection power was averaged over the 12 possible years of experiment for the OptiMET MET, the expert MET, and the 120 random METs. A QTL was considered to be detected if at least one SNP located at a maximum distance of 1 cM had a *p* value below the threshold 0.05/25

METs	VAI	SLDL	Phyl
OptiMET	16.7	18.0	10.7
Expert	13.0	11.7	4.2
Random	12.6	10.1	3.0

#### Prediction accuracy of the parameter values of individuals in validation obtained with the different METs

**Fig. 5:**
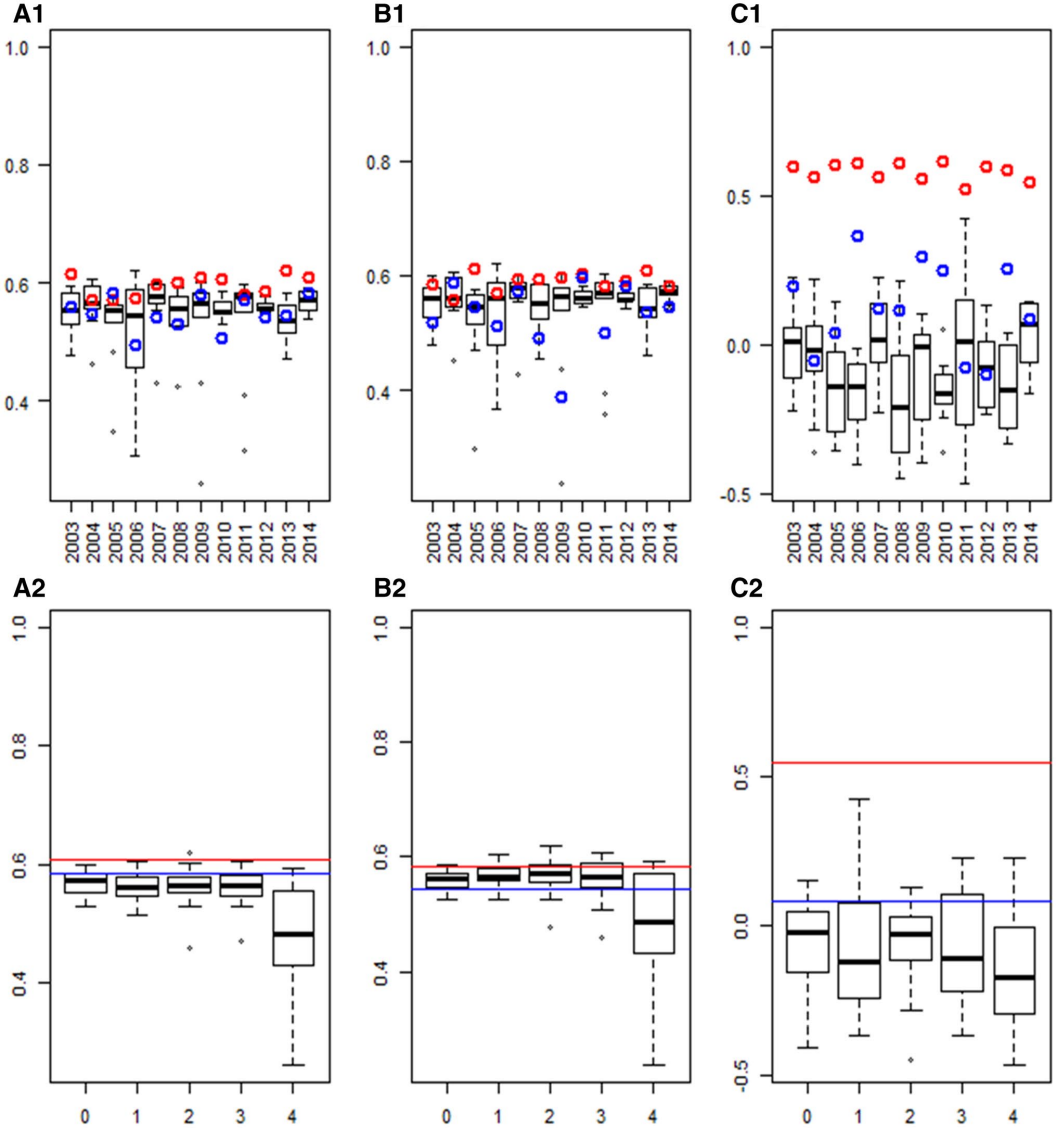
Prediction accuracy of the parameter values of the individuals in validation. The GS model was calibrated with the parameter estimates resulting from the different METs: 120 randomly sampled METs (*boxplots*), one expert MET (*blue points* for specific years and *blue lines* for the average across years), and the OptiMET MET (*red points* for specific years and *red lines* for the average across years). The results are presented for each of the 12 years of experiment (**a1**, **b1,** and **c1**), or for each of the random sampling strategy (**a2**, **b2,** and **c2**, number of winter sowings on the *x axis*), for VAI **a**, SLDL **b,** and Phyl **c** (color figure online)

The parameter estimates resulting from the different METs were then used to calibrate a GS model and predict the parameter values of the individuals in validation (Fig. 5). Prediction accuracies were variable between parameters, between years and between METs. On average, prediction accuracies were higher for SLDL and VAI than for Phyl. On average, the different random sampling strategies resulted in similar accuracies for VAI and SLDL, except when the four winter sowings were sampled, which resulted in lower accuracies (Fig. 5a2, b2). On average, the OptiMET MET performed better than the other strategies, particularly for Phyl for which accuracy was on average multiplied by about three compared to expert and random METs.

#### Prediction accuracy and RMSE of heading time of individuals in validation in independent environments

**Fig. 6:**
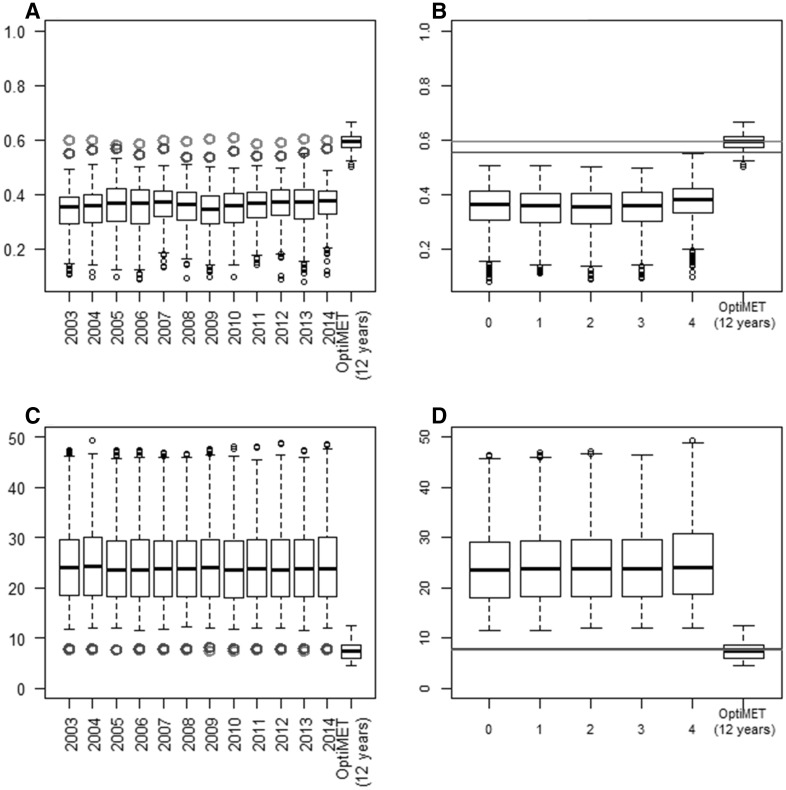
Prediction accuracy (**a**, **b**) and root-mean-square error (RMSE, **c**, **d**) of heading time for the individuals in validation. Heading time was predicted using the predictions of the parameters of the individuals in validation as input for the crop model. The parameter estimates of the calibration set were obtained with different METs: 120 randomly sampled METs (*boxplots*), one expert MET (*blue points* for specific years and *blue line* for the average across years), and the OptiMET MET (*red points* for specific years and* red line* for the average across years). The results are presented for each of the 12 years of experiment (**a**, **c**), or for each of the random sampling strategy (**b**, **d**; number of winter sowings on the *x axis*) (color figure online)

The last step was to use the parameter predictions of the 270 individuals in validation to predict their heading time in 28 independent environments using the CGM. The prediction accuracies and RMSE of heading time resulting from the different METs used to estimate the parameters were computed (Fig. 6). Prediction accuracy was higher with the OptiMET MET (0.59) than with the expert (0.55) and random METs (0.35), on average. The gain brought by OptiMET varied greatly between years (in 2009, OptiMET performed much better than the expert MET, but similarly for year 2005). The best random sampling strategy was to sample four winter sowings (Fig. 6b), which can be explained by the absence of spring sowing in the validation data set composed of winter sowing environments only. The difference between the OptiMET and the expert MET was less pronounced when looking at the RMSE (Fig. 6c, d), but OptiMET performed better than the expert MET in 97% of the cases.

#### Composition of the OptiMET MET

**Fig. 7:**
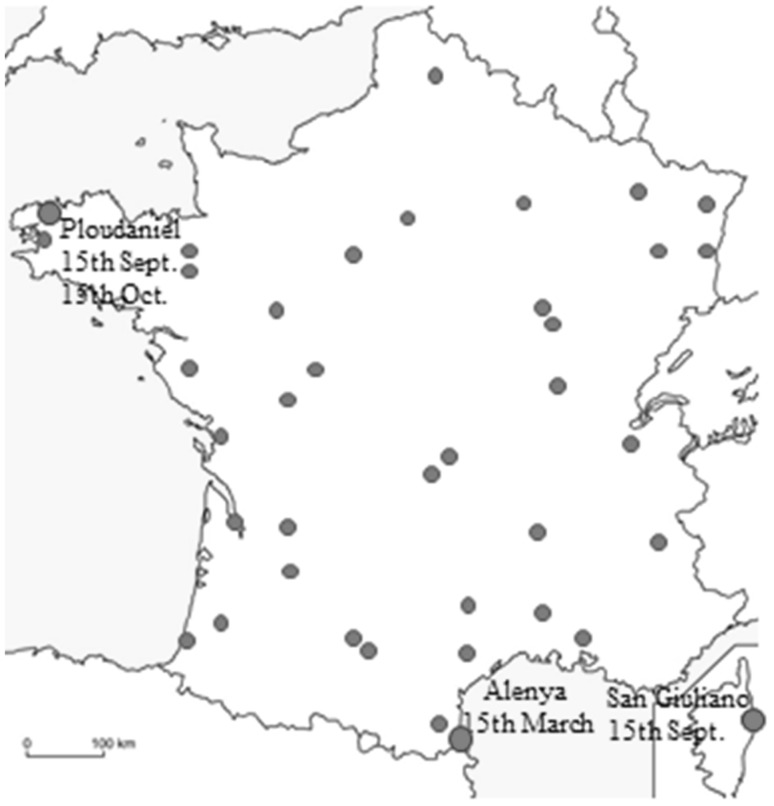
MET sampled by OptiMET. The locations sampled by OptiMET are visualized by *red dots*. *Sowing dates* are indicated near the sampled locations (color figure online)

The OptiMET MET (Fig. 7) is composed of two locations in the south of France (Alenya and San Giuliano) and one location in the west (Ploudaniel). There were three sowing dates in winter and one in spring. Alenya and San Giuliano are under Mediterranean conditions, whereas Ploudaniel is submitted to oceanic climate. The three locations have mild winters.

### Evaluation of OptiMET on a real data set

**Fig. 8:**
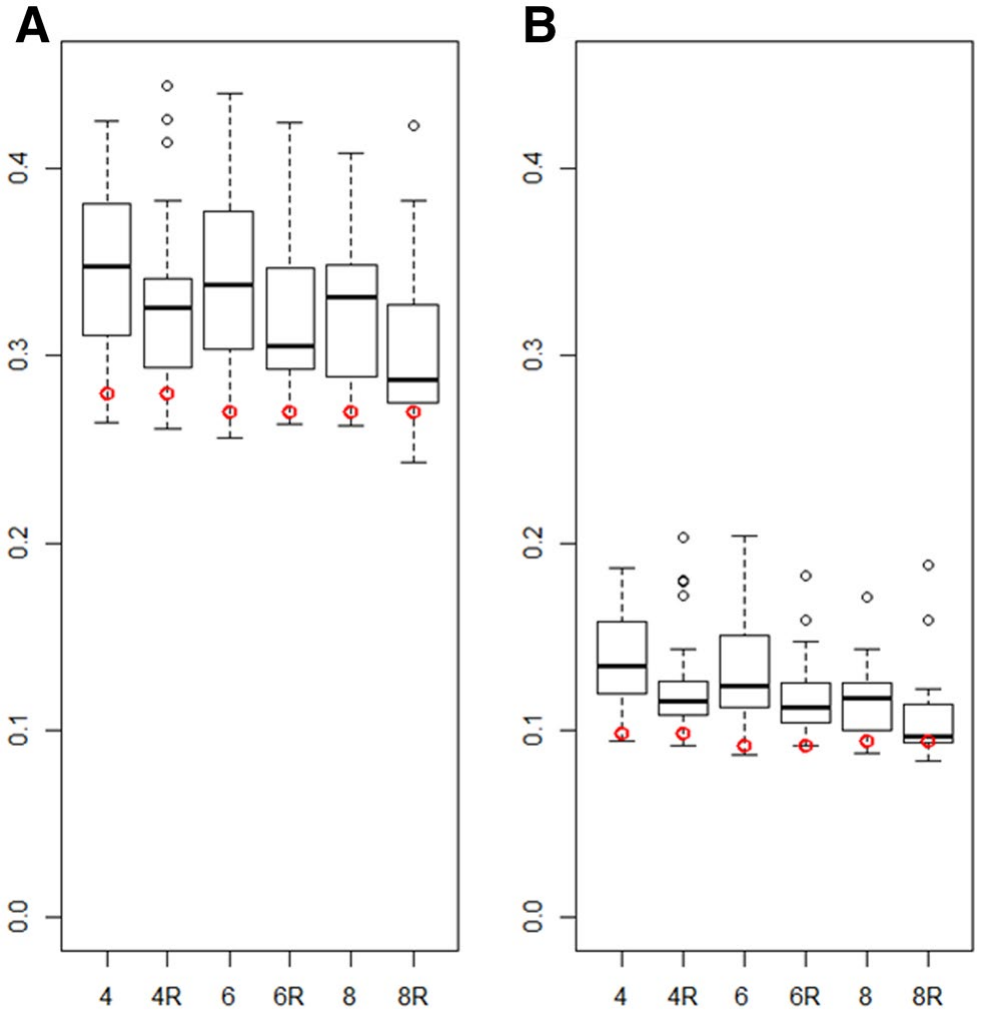
Normalized RMSE and normalized PSE averaged over the three parameters (**a** $\overline{{\text{NRMSE}^{\text{*}} }}$, **b** $\overline{{\text{NPSE}}}$) of random METs and of reasoned METs composed of 4, 6, or 8 environments (denoted by 4, 6, and 8 and 4R, 6R, and 8R, respectively). Each boxplot is composed of 40 METs. The $\overline{{\text{NRMSE}^{\text{*}} }}$ and $\overline{{\text{NPSE}}}$ obtained with the OptiMET MET of the corresponding number of trials is represented by a *red dot*

The criteria ($\overline{{{\text{NRMSE}}^{*} }}$ and $\overline{\text{NPSE}}$) used to evaluate the different METs were highly variable for the random samples (Fig. 8). As expected, $\overline{{{\text{NRMSE}}^{*} }}$ and $\overline{\text{NPSE}}$ decreased when the size of the METs increased. The reasoned METs were on average more efficient than random METs. The OptiMET MET performed better than the random and reasoned METs on average, and performed similarly than the best random and reasoned METs. The difference of $\overline{{{\text{NRMSE}}^{*} }}$ or $\overline{\text{NPSE}}$ between the average of the random (and reasoned) METs and the OptiMET MET decreased when the size of the experimental design increased, which was expected, because by construction, the overlap between random and OptiMET METs increases with the size of the METs. According to both criteria, the OptiMET MET composed of four trials performed better than the reasoned MET composed of eight trials on average ($\overline{{{\text{NRMSE}}^{ *} }}$ of 0.28 and 0.29 for the OptiMET MET of size 4 and for the average of the reasoned METs of size 8, respectively).

## Discussion and conclusions

The interest of using ecophysiological modelling to better model GEI is now well recognized in the plant genetics community (Chapman et al. 2002; Hammer et al. 2002; Reymond et al. 2003; Heslot et al. 2014; Technow et al. 2015; Bustos-Kort et al. 2016). It has been shown in various studies that CGM could be used to structure environments in groups according to the type and frequency of stress experienced by the crop (Löffler et al. 2005; Hammer and Jordan 2007; Chenu et al. 2011). This clustering approach has the advantage of reducing GEI within each group of environments, which facilitates the implementation of GS.

In the present study, a more integrated approach was applied: CGM was used to characterize varieties by genetic parameters expected to be independent from the environment. This means that the QTL detected for these traits are stable across environments, and their prediction accuracy will also be independent from the environments. Once the QTL are detected and the GS model calibrated, it is possible to predict the values of these traits for various varieties, which can then be used to predict their performances in various potentially new or virtual environments thanks to the CGM. This approach (CGM-MAS) is potentially highly powerful but relies on a difficult task which is the estimation of the genetic parameters. Moreover, CGM-MAS is composed of many successive steps, probably leading to error propagation. As a result, to run CGM-MAS efficiently, one has to optimize each step of the process, in particular at first the estimation of the parameters which affects all the following steps. Here, we propose a criterion called OptiMET to define an optimal set of environments (MET) for the estimation of the genetic parameters, i.e., an MET generating parameter estimates with low error variance. OptiMET was inspired from a study on hydrological modelling (Leube et al. 2012) in the context of Bayesian model averaging. OptiMET was tested using simulations and a real data set, with the example of wheat phenology.

### Evaluation of the criterion OptiMET using simulations

The NRMSE of the parameter estimates were clearly variable between years of experiments and between parameters (Fig. 3). The year effect was large and affected both the average and the variability of the NRMSE, which can be explained by the important year effect on climatic conditions. This year effect was for example important for the OptiMET MET, which NRMSE could almost double from one year to another (for example 2007 and 2008 for VAI, Fig. 3). This year effect affected the three parameters differently, because they are not influenced by the same environmental covariates. It is interesting to note that the NRMSE of SLDL, which is affected by an environmental covariate stable across years (day length), also showed between year variability, revealing the complex dependencies between parameters. However, despite these variabilities, the ranking of the sampling approaches remained the same, with OptiMET doing better than the expert and the random METs. The difference between the NRMSE of the OptiMET MET and the average NRMSE of the random METs varied between years, but OptiMET did always better or as good as the best random METs for the three parameters. The expert MET unexpectedly performed poorly, doing sometimes better, sometimes worse than random samples, and was particularly inefficient to estimate the parameter Phyl. This could be explained by the fact that in the location x sowing date, combinations composing the expert MET Phyl did not participate much in the variability of heading time as revealed by sensitivity analysis (results not shown). For the random METs, it appeared that sampling both winter and spring sowing dates performed better, doing best when two or three winter sowings were sampled (Fig. 3a2, b2). These combinations of winter and spring sowings are, indeed, supposed to decorrelate the effect of the different parameters, and that is the reason why the combination “three winter sowings and one spring sowing” was chosen in the expert MET.

Similar conclusions could be drawn on detection power (Fig. 4), as it is influenced by the error variance (and thus by NRMSE). One main conclusion, common to all METs, is that detection power was low for the three parameters with a maximum of 18% for SLDL with OptiMET. This could be explained by the fact that the simulated genetic architecture was influenced by 25 QTL following geometric series (Lande and Thompson 1990), which means that most of these QTL explained a small portion of the total genetic variance. For some real traits, major QTL can exist and have thus to be taken into account in the construction of the prediction formula. In our case, as many QTL were simulated (25 for each parameter), predictions were made with a classical G-BLUP model. Prediction accuracy of the parameter values of 270 independent varieties (Fig. 5) was also variable between years and METs, but again, OptiMET MET performed better than other METs with accuracies always above 0.52.

When these parameter predictions were used to predict heading time in independent MET using the CGM, the prediction accuracies obtained were high for the OptiMET MET and the expert MET each year (around 0.6, Fig. 6). Although the difference of efficiency between OptiMET and the expert MET was less pronounced than with the parameter values accuracies, OptiMET always did better than the expert MET and was more stable across years. For some years, the difference between the OptiMET and the expert MET was, indeed, more important (for example 2003, 2008 and 2009), probably because heading time was more sensitive to Phyl variations for these years. Prediction accuracies of the random METs were on average much lower and sometimes negative, and this time, the METs composed of four winter sowings performed on average better than the other combinations. This could be explained by the fact that the validation environments were all winter sowings (representing actual agricultural practices), and thus, METs composed of four winter sowings are more representative of what happens in the validation environments. It is interesting to note that the OptiMET MET which is composed of three winter sowings (and one spring sowing) performed better than random METs composed of four winter sowings.

### Evaluation of OptiMET on real data

Working on simulated data sets is interesting, because we know the truth and we can generate various situations, but it is often simplistic in comparison to real experiments. We, therefore, compared the efficiency of various real METs for the parameter estimation. As expected, the NRMSE obtained with METs of the same size (four location × sowing date combinations) were higher for the real data set than for the simulated data set (Figs. 3, 8). With the real data set, the quality criteria ($\overline{{{\text{NRMSE}}^{ *} }}$ and $\overline{\text{NPSE}}$) decreased with the size of the MET, but this decrease was slow (Fig. 8). This difference between the real and the simulated data sets can be explained by the fact that there were 156 simulated environments, whereas only 26 real environments and the simulated environments were much more variable in comparison to the 26 real environments (more than half of these 26 environments were October or November sowings in the North of France). In addition to this, it is possible that the heritabilities of the 26 environments were lower than the heritability simulated in the first part. Unfortunately, there were no sufficient observations (no repetitions) to estimate the heritabilities in the 26 environments. Another point is that $\overline{{{\text{NRMSE}}^{ *} }}$ and $\overline{\text{NPSE}}$ are computed using the parameter estimates obtained with the 26 environments and are thus also subjected to estimation errors. However, the METs sampled with OptiMET were always among the most efficient, and the OptiMET MET composed of four location x sowing date combinations performed similarly than the quantile of the best random and reasoned METs of size eight. One point that may have limited the efficiency of OptiMET with this data set is that the meteorological data used to compute OptiMET were obtained from the meteorological stations, the closest from the location, which was sometimes a few kilometers away. To compute OptiMET more efficiently, it would have been necessary to measure daily temperature at each location of experiment.

We can conclude from this part, that with this real data set which was specifically produced to study the phenological parameters, choosing the best eight environments lead to parameter estimates of poor quality (in comparison to the simulated data sets). Even more contrasted environments are required. However, one major conclusion is that OptiMET was efficient to define better METs, which means that it is an interesting tool to determine experimental design before collecting phenotypes.

### Limits and perspectives of OptiMET

We have shown here that in the context of CGM-MAS, experimental designs could be optimized before having access to any observation. However, we have to keep in mind that the use of OptiMET requires (1) a robust CGM for the trait of interest in the considered environments, (2) environmental covariates that can be predicted or at least approximated before doing the experiment, and (3) prior knowledge on the distribution of each genetic parameter (at least the bounds of the distribution). For (1), we can benefit from decades of ecophysiological research which resulted in the development of reference CGM such as SIRIUS, APSIM, STICS, or CERES which simulate the development of the plant from sowing to yield elaboration (Martre et al. 2015b; Yin and Struik 2016). However, each model has its specificities and its own domain of validity, which means that their predictions are reliable in some ranges of environments. Therefore, when using OptiMET to define optimal experimental design (and more generally to lead CGM-MAS approaches), we have to make sure that the chosen environments are in the range of validity of the CGM. This is an important point to consider to use OptiMET efficiently, because this criterion will by definition identify contrasted environments. Therefore, one has to take care that the possible environments proposed to OptiMET are all in the range of validity of the CGM. The METs sampled by OptiMET result in contrasted phenotypes, which means that the phenotypes may be more difficult to measure. With the example of wheat phenology, spring sowings result in heading time spread across a period of few weeks to few months, and so, it will be more difficult for the experimenter to follow the plant development day by day over this long period. A special care has to be put on these experiments to reach high heritabilities. For (2), we have shown that average climatic data could be used to approximate the climate of future years for the CGM that we have considered. However, this will certainly not be true for all CGM, particularly if they rely on more erratic covariates as rainfall. In that case, it might be useful to use climate generators such as LARS (Semenov et al. 1998) and to take into account the inter-year variability when computing OptiMET. In such context, one can compute OptiMET for many specific years (using the climatic conditions of past years or simulated years) and choose the optimal MET which leads to low OptiMET values across years. Of course, if the experiment can be done in controlled conditions such as high-throughput phenotyping platforms, the use of OptiMET will be much easier and will allow to tune the covariates that can be controlled on these platforms. The third point that has to be taken care of (3) is the definition of the distribution of the genetic parameters. Prior knowledge on the distribution of each genetic parameter is, indeed, necessary to compute OptiMET (at least the bounds of the distributions). Here, we had no more information than the minimal and maximal values of each parameter (defined by expert knowledge and/or literature), so the values were chosen to get a uniform coverage of the parameters space. However, if more information is available, it would be possible to improve these distributions, for example by taking into account that some values are more probable than others, or that some genetic parameters are correlated. The more information is available on the joint distributions of the genetic parameters, the more realistic will be the values sampled, and the more efficient will be OptiMET. Indeed, if the values are chosen according to these informative distributions, then OptiMET will automatically put more weight on the parts of the parameter space which are more probable to occur.

Alternative uses of OptiMET that were not illustrated in this paper are the definition of METs to estimate efficiently one or few specific parameters (instead of a full parameterization as performed in the present work). Such an approach would be relevant for example when a focus is made on the genetic architecture of a specific parameter. Another potential use would be to define METs that are complementary to already existing data sets. When studying the effect of abiotic stress in multi-local trials, it often happens that the experiments do not cover the whole range of stress that was expected. In such a situation, it could be valuable to use criteria such as OptiMET to define additional complementary experiments (in controlled conditions and/or in a minimal but optimal MET) with for example stress scenarios which were missing in the existing data set.

The present study illustrated that OptiMET could be efficient to determine optimal experimental design of a given size (number of location x sowing date combinations), but using OptiMET to define an optimal size of experimental design would be more complicated. In other words, OptiMET is efficient to compare experimental designs of the same size, but not to estimate a risk (a level of precision) associated to these experimental designs. Further methodological developments are required for this.

In conclusion, the data sets studied here clearly showed that choosing relevant experimental designs was highly important to lead CGM-MAS approaches. The quality of the parameter estimates, indeed, influences all the following steps of the CGM-MAS process, including the performance predictions. The criterion OptiMET was efficient to define such optimal experimental designs and resulted in better parameter estimates both on simulated and real data. It would be now interesting to evaluate this criterion on other traits simulated by other CGM.

#### Author contribution statement

Conceived and designed the experiments: RR, EK, VA, JLG, HM, FXO, and MR. Analyzed the data: RR. Wrote the paper: RR. Revised the manuscript critically: EK, VA, JLG, and HM.

## Electronic supplementary material

Below is the link to the electronic supplementary material.

Supplementary material 1 (DOCX 217 kb)
