# Supplementary material for: Optimization of multi-environment trials for genomic selection based on crop models
Source: Theor Appl Genet. 2017 May 24;130(8):1735–52. doi: 10.1007/s00122-017-2922-4 (PMC5511605; doi:10.1007/s00122-017-2922-4)
Supplement: Supplementary file 1 — Supplementary material 1 (DOCX 217 kb) [file 122_2017_2922_MOESM1_ESM.docx]

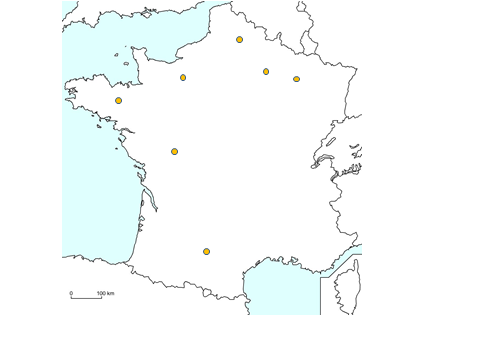

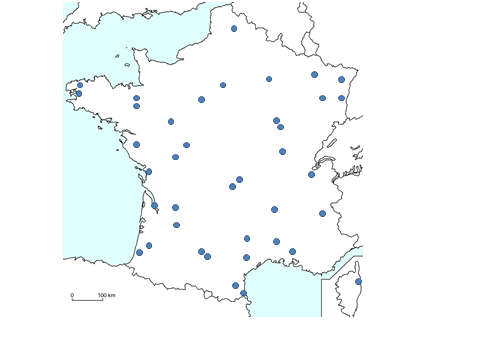


Figure S1: Locations considered in the simulation study. Left: the 39 possible locations available to compose the multi-environment trials. Right: The seven independent locations used for validation.
